# Supplementary material for: Synergistic Effects of Anthocyanin-Enriched Morus alba L. Extract and Vitamin C: Promising Nutraceutical Ingredients in Functional Food Development for Neuroprotection
Source: Foods. 2025 Oct 24;14(21):3630. doi: 10.3390/foods14213630 (PMC12610446; doi:10.3390/foods14213630)
Supplement: Supplementary file 1 [file foods-14-03630-s001.zip › foods-3936406-supplementary.pdf]

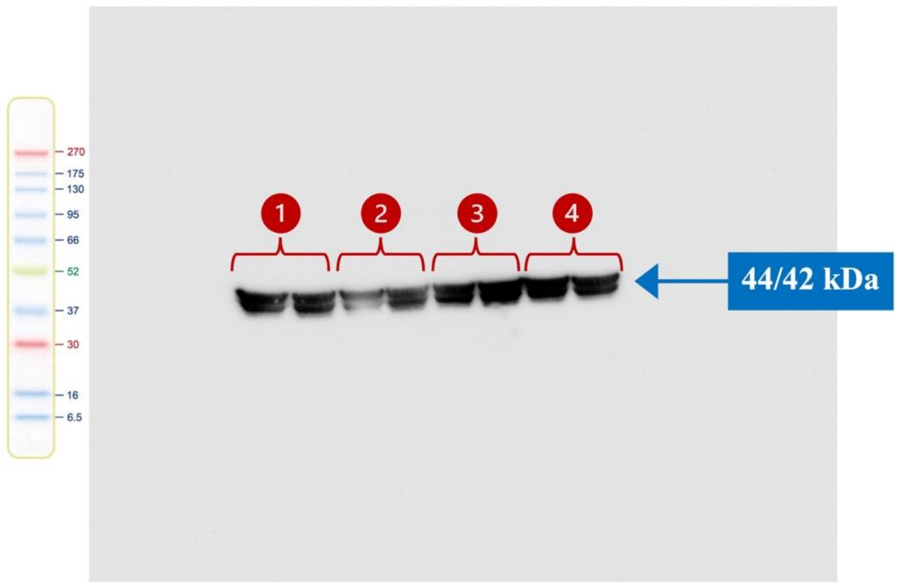

Figure S1: Western blot analysis of p-ERK expression in SH-SY5Y cells following treatment with MAC against hydrogen peroxide-induced neurotoxicity. The experimental groups were: (1) control, (2) hydrogen peroxide + vehicle, (3) hydrogen peroxide + MAC1, and (4) hydrogen peroxide + MAC2. MAC1: anthocyanin-enriched *Morus alba* L. extract combined with vitamin C at 1X:1X; MAC2: anthocyanin-enriched *Morus alba* L. extract combined with vitamin C at 1X:0.5X. This figure shows the original, unprocessed, uncropped, full-length membrane images.

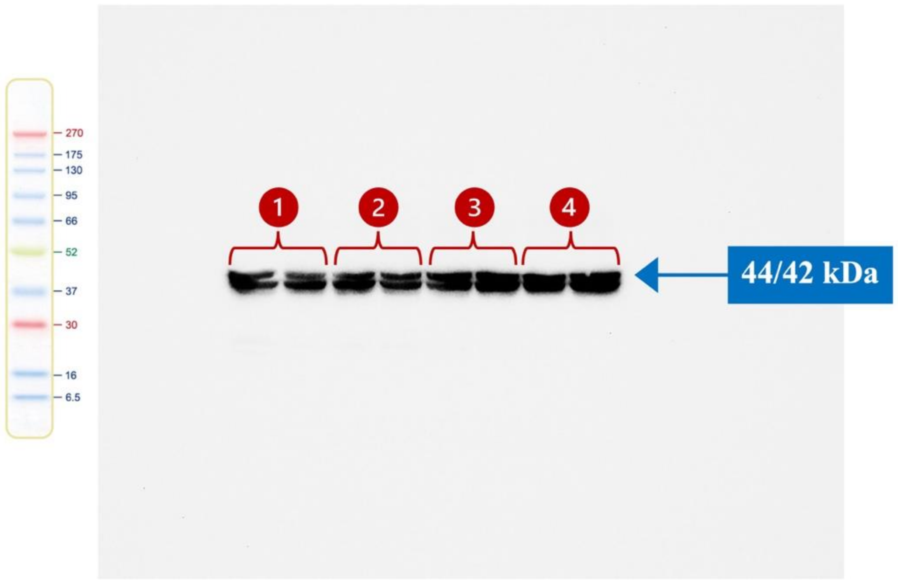

Figure S2: Western blot analysis of ERK expression in SH-SY5Y cells following treatment with MAC against hydrogen peroxide-induced neurotoxicity. The experimental groups were: (1) control, (2) hydrogen peroxide + vehicle, (3) hydrogen peroxide + MAC1, and (4) hydrogen peroxide + MAC2. MAC1: anthocyanin-enriched *Morus alba* L. extract combined with vitamin C at 1X:1X; MAC2: anthocyanin-enriched *Morus alba* L. extract combined with vitamin C at 1X:0.5X. This figure shows the original, unprocessed, uncropped, full-length membrane images.

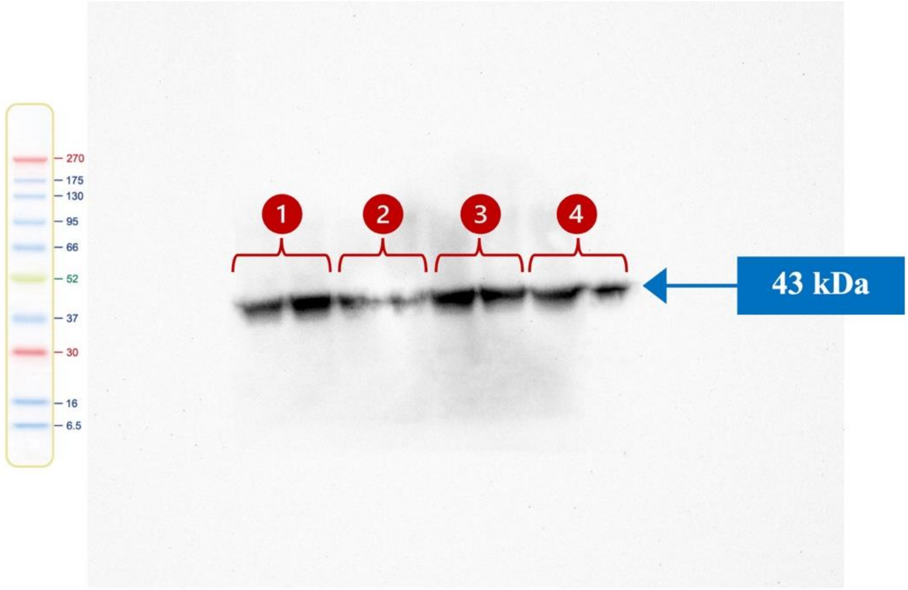

Figure S3: Western blot analysis of CREB expression in SH-SY5Y cells following treatment with MAC against hydrogen peroxide-induced neurotoxicity. The experimental groups were: (1) control, (2) hydrogen peroxide + vehicle, (3) hydrogen peroxide + MAC1, and (4) hydrogen peroxide + MAC2. MAC1: anthocyanin-enriched *Morus alba* L. extract combined with vitamin C at 1X:1X; MAC2: anthocyanin-enriched *Morus alba* L. extract combined with vitamin C at 1X:0.5X. This figure shows the original, unprocessed, uncropped, full-length membrane images.

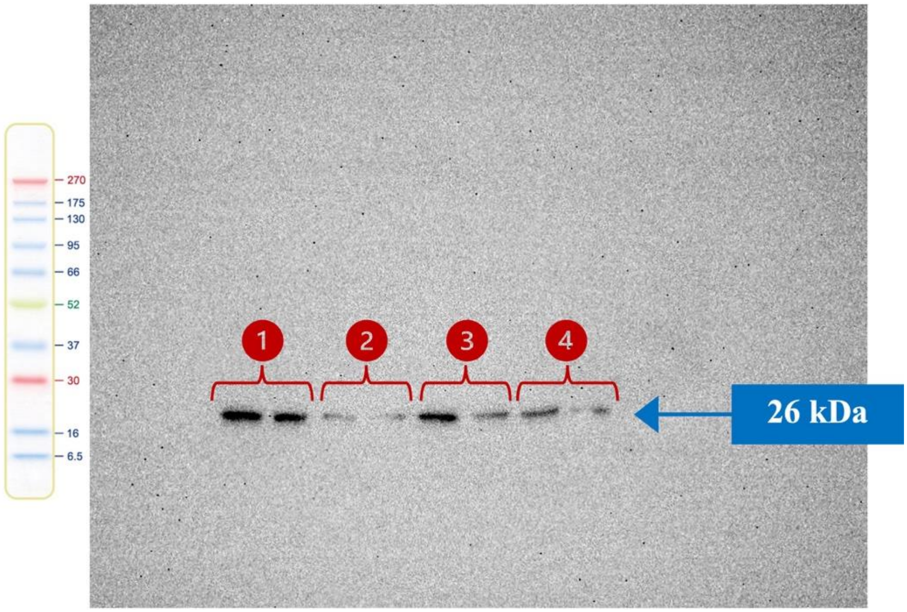

Figure S4: Western blot analysis of Bcl-2 expression in SH-SY5Y cells following treatment with MAC against hydrogen peroxide-induced neurotoxicity. The experimental groups were: (1) control, (2) hydrogen peroxide + vehicle, (3) hydrogen peroxide + MAC1, and (4) hydrogen peroxide + MAC2. MAC1: anthocyanin-enriched *Morus alba* L. extract combined with vitamin C at 1X:1X; MAC2: anthocyanin-enriched *Morus alba* L. extract combined with vitamin C at 1X:0.5X. This figure shows the original, unprocessed, uncropped, full-length membrane images.

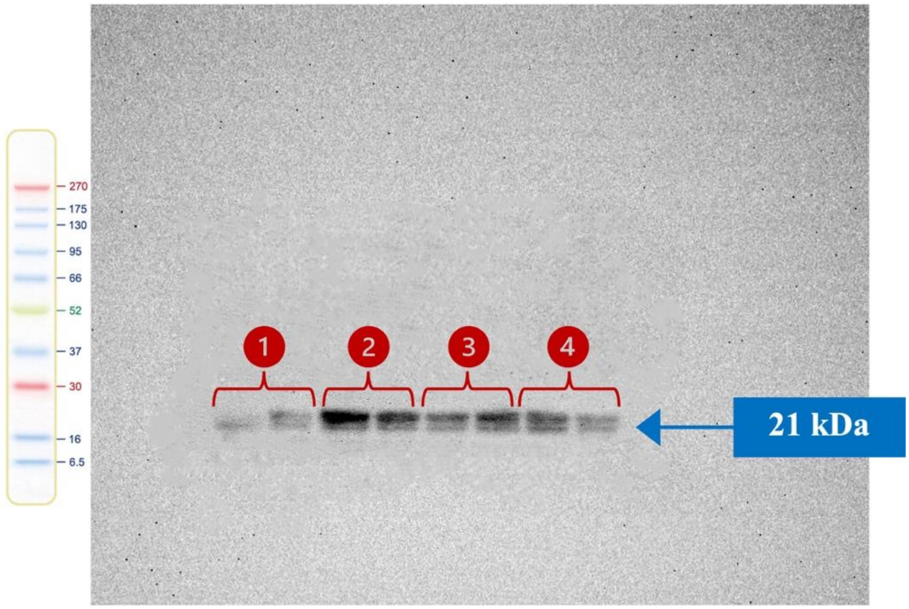

Figure S5: Western blot analysis of BAX expression in SH-SY5Y cells following treatment with MAC against hydrogen peroxide-induced neurotoxicity. The experimental groups were: (1) control, (2) hydrogen peroxide + vehicle, (3) hydrogen peroxide + MAC1, and (4) hydrogen peroxide + MAC2. MAC1: anthocyanin-enriched *Morus alba* L. extract combined with vitamin C at 1X:1X; MAC2: anthocyanin-enriched *Morus alba* L. extract combined with vitamin C at 1X:0.5X. This figure shows the original, unprocessed, uncropped, full-length membrane images.

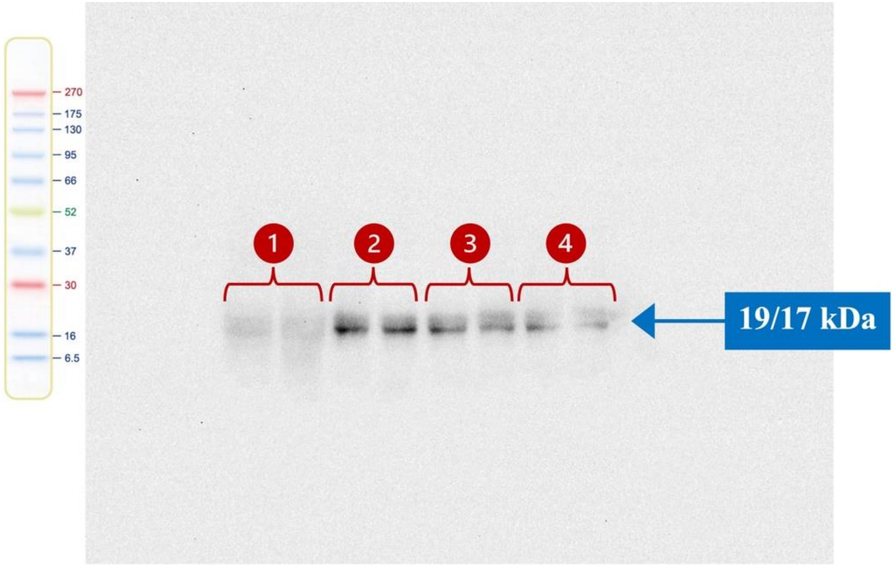

Figure S6: Western blot analysis of cleaved caspase-3 expression in SH-SY5Y cells following treatment with MAC against hydrogen peroxide-induced neurotoxicity. The experimental groups were: (1) control, (2) hydrogen peroxide + vehicle, (3) hydrogen peroxide + MAC1, and (4) hydrogen peroxide + MAC2. MAC1: anthocyanin-enriched *Morus alba* L. extract combined with vitamin C at 1X:1X; MAC2: anthocyanin-enriched *Morus alba* L. extract combined with vitamin C at 1X:0.5X. This figure shows the original, unprocessed, uncropped, full-length membrane images.

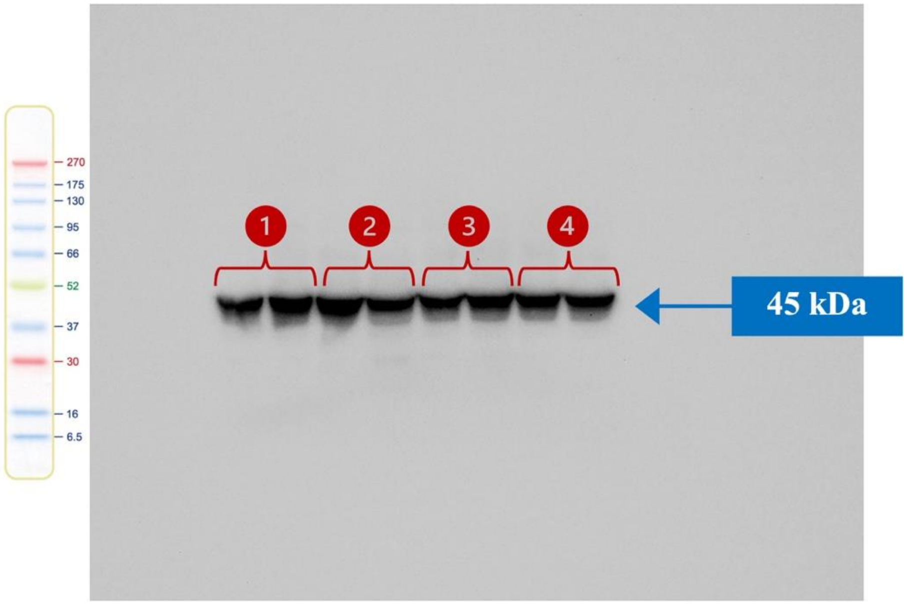

Figure S7: Western blot analysis of **β**-actin expression in SH-SY5Y cells following treatment with MAC against hydrogen peroxide-induced neurotoxicity. The experimental groups were: (1) control, (2) hydrogen peroxide + vehicle, (3) hydrogen peroxide + MAC1, and (4) hydrogen peroxide + MAC2. MAC1: anthocyanin-enriched *Morus alba* L. extract combined with vitamin C at 1X:1X; MAC2: anthocyanin-enriched *Morus alba* L. extract combined with vitamin C at 1X:0.5X. This figure shows the original, unprocessed, uncropped, full-length membrane images.

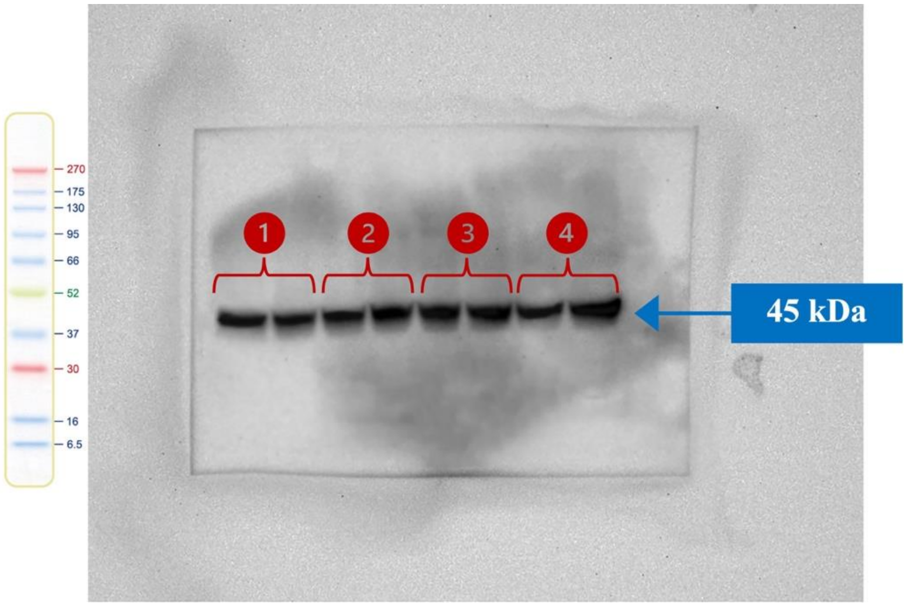

Figure S8: Western blot analysis of **β**-actin expression in SH-SY5Y cells following treatment with MAC against hydrogen peroxide-induced neurotoxicity. The experimental groups were: (1) control, (2) hydrogen peroxide + vehicle, (3) hydrogen peroxide + MAC1, and (4) hydrogen peroxide + MAC2. MAC1: anthocyanin-enriched *Morus alba* L. extract combined with vitamin C at 1X:1X; MAC2: anthocyanin-enriched *Morus alba* L. extract combined with vitamin C at 1X:0.5X. This figure shows the original, unprocessed, uncropped, full-length membrane images.

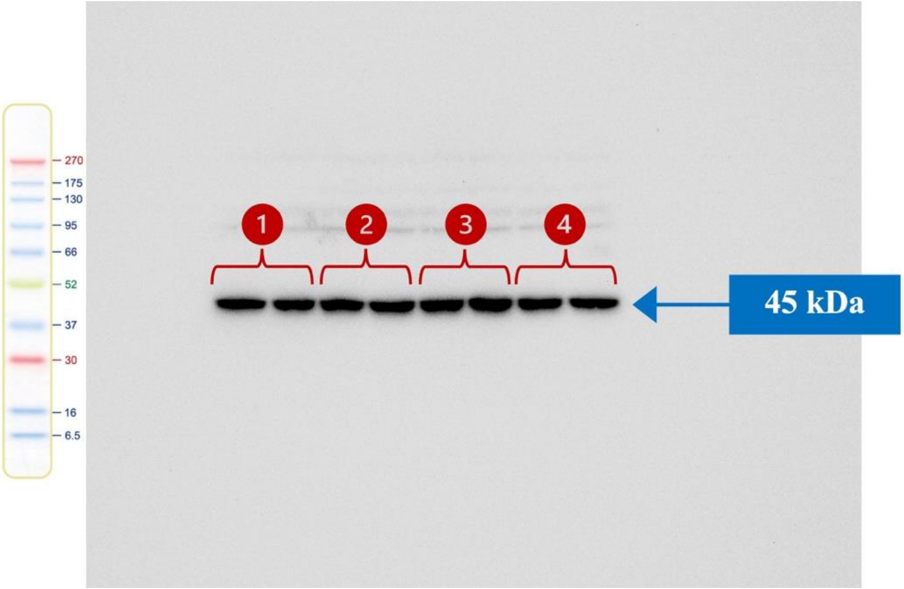

Figure S9: Western blot analysis of **β**-actin expression in SH-SY5Y cells following treatment with MAC against hydrogen peroxide-induced neurotoxicity. The experimental groups were: (1) control, (2) hydrogen peroxide + vehicle, (3) hydrogen peroxide + MAC1, and (4) hydrogen peroxide + MAC2. MAC1: anthocyanin-enriched *Morus alba* L. extract combined with vitamin C at 1X:1X; MAC2: anthocyanin-enriched *Morus alba* L. extract combined with vitamin C at 1X:0.5X. This figure shows the original, unprocessed, uncropped, full-length membrane images.

Table S1: Original input data for dose–response and synergy analysis of anthocyanin-enriched *Morus alba* L. extract and vitamin C

PairIndex	Drug1	Drug2	Conc1	Conc2	Response	ConcUnit
1	*Morus alba* L. extract	Vitamin C	2.6625	0.0000	34.85	µg/mL
1	*Morus alba* L. extract	Vitamin C	5.3250	0.0000	41.85	µg/mL
1	*Morus alba* L. extract	Vitamin C	10.6500	0.0000	50.61	µg/mL
1	*Morus alba* L. extract	Vitamin C	21.3000	0.0000	55.41	µg/mL
1	*Morus alba* L. extract	Vitamin C	42.6000	0.0000	70.66	µg/mL
1	*Morus alba* L. extract	Vitamin C	2.6625	5.4125	49.98	µg/mL
1	*Morus alba* L. extract	Vitamin C	5.3250	5.4125	58.89	µg/mL
1	*Morus alba* L. extract	Vitamin C	10.65900	5.4125	77.64	µg/mL
1	*Morus alba* L. extract	Vitamin C	21.3000	5.4125	79.71	µg/mL
1	*Morus alba* L. extract	Vitamin C	42.6000	5.4125	82.65	µg/mL
1	*Morus alba* L. extract	Vitamin C	2.6625	10.8250	54.69	µg/mL
1	*Morus alba* L. extract	Vitamin C	5.3250	10.8250	61.69	µg/mL
1	*Morus alba* L. extract	Vitamin C	10.6500	10.8250	79.09	µg/mL
1	*Morus alba* L. extract	Vitamin C	21.3000	10.8250	81.51	µg/mL
1	*Morus alba* L. extract	Vitamin C	42.6000	10.8250	79.11	µg/mL
1	*Morus alba* L. extract	Vitamin C	2.6625	21.6500	56.85	µg/mL
1	*Morus alba* L. extract	Vitamin C	5.3250	21.6500	64.06	µg/mL
1	*Morus alba* L. extract	Vitamin C	10.6500	21.6500	88.80	µg/mL
1	*Morus alba* L. extract	Vitamin C	21.3000	21.6500	90.15	µg/mL
1	*Morus alba* L. extract	Vitamin C	42.6000	21.6500	79.69	µg/mL
1	*Morus alba* L. extract	Vitamin C	2.6625	43.3000	58.78	µg/mL
1	*Morus alba* L. extract	Vitamin C	5.3250	43.3000	69.96	µg/mL
1	*Morus alba* L. extract	Vitamin C	10.6500	43.3000	82.55	µg/mL
1	*Morus alba* L. extract	Vitamin C	21.3000	43.3000	91.34	µg/mL
1	*Morus alba* L. extract	Vitamin C	42.6000	43.3000	81.28	µg/mL
1	*Morus alba* L. extract	Vitamin C	2.6625	86.6000	55.78	µg/mL
1	*Morus alba* L. extract	Vitamin C	5.3250	86.6000	68.40	µg/mL
1	*Morus alba* L. extract	Vitamin C	10.6500	86.6000	76.54	µg/mL
1	*Morus alba* L. extract	Vitamin C	21.3000	86.6000	80.36	µg/mL
1	*Morus alba* L. extract	Vitamin C	42.6000	86.6000	79.38	µg/mL
1	*Morus alba* L. extract	Vitamin C	0.0000	5.4125	32.18	µg/mL
1	*Morus alba* L. extract	Vitamin C	0.0000	10.8250	39.64	µg/mL
1	*Morus alba* L. extract	Vitamin C	0.0000	21.6500	50.21	µg/mL
1	*Morus alba* L. extract	Vitamin C	0.0000	43.3000	70.11	µg/mL
1	*Morus alba* L. extract	Vitamin C	0.0000	86.6000	54.00	µg/mL
1	*Morus alba* L. extract	Vitamin C	0.0000	0.0000	0.00	µg/mL
